# On the grammar of restricted-range numeral systems

**DOI:** 10.1098/rstb.2024.0213

**Published:** 2025-10-20

**Authors:** Claire Bowern

**Affiliations:** ^1^ Department of Linguistics, Yale University, New Haven, CT 06511, USA

**Keywords:** numerals, bases, quantification, cognition

## Abstract

Languages lexicalize numerical concepts in many ways. Here I focus on what so-called ‘low-limit’ or ‘restricted-range’ numeral systems in Australian languages tell us about the linguistics of number concepts and how they change over time. It is a given in contemporary cognitive science that the types of operations that are available cognitively are human universals. But it is also obvious that languages and cultures differ in what ends up expressed in words and grammar. Numerals are one such area of variation. Australian restricted-range numerals come from the same sources as numerals elsewhere. Semantically, they appear to behave similarly to productive numerals. Australian restricted-range systems do not appear to behave like morphological number, as has sometimes been claimed. Why such systems are restricted is unclear; several hypotheses are investigated.

This article is part of the theme issue ‘A solid base for scaling up: the structure of numeration systems’.

## 1. Introduction

This article is about the way that languages lexicalize numerical concepts, focusing on what so-called ‘low-limit’ or ‘restricted-range numeral systems reveal about the linguistics of number concepts, especially their systemic composition and their use in grammar. It is a given in modern cognitive science and anthropology that the types of operations that are available cognitively are human universals. But it is also obvious that languages and cultures differ in what ends up expressed in words and what is brought into grammatical systems. Some aspects of human cognition are never grammaticalized: the ability to perceive three-dimensional objects is a human universal but never part of grammar. Colour is lexicalized in different ways across languages [1] but there are no grammatical inflections in human languages that index colour. Other concepts are universally lexicalized but seldom grammaticalized: words for kin are universal [2] but systems that incorporate kinship into pronouns or other aspects of grammar are rare [3–5]. Quantification concepts, however, are instantiated across both the lexicon and the grammar. In the lexicon, there are numerals, such as *one* and *two*, while in the grammar, there is number marking, e.g. dual and plural. Quantification and measurement crosscut lexicon and grammar, as discussed in more detail below. This means that numerals are embedded in a syntactic system that also includes measurement, comparison of degrees and quantities (e.g. *more than*), and part of understanding and comparing numeral words is learning how they interact with morphology, syntax and semantics.

Cross-linguistically, numeral ranges may be unrestricted or restricted, with numerals having an upper limit (usually below 10; cf. [6]). An open question is whether numerals in restricted systems have the same syntax as unrestricted numerals (or rather, how they fit in numeral variation cross-linguistically). In this article, I specifically examine what the restricted-range systems of Australian Indigenous languages can tell us about numerical concepts and how they work in grammar.^1^ I ask three questions:

(1) Do restricted-range numeral systems come from the same sources as unrestricted systems?
(2) Do restricted-range systems behave grammatically and semantically like unrestricted systems, or are there other differences other than the limit of the range of numerical expression? That is, what does research on unrestricted systems help us understand about restricted-range systems and vice versa?
(3) What do restricted-range systems tell us about anchors, compositionality and productivity in linguistic numeral systems?

Restricted-range numeral systems in Australia have usually been described through a ‘deficit lens’—that is, descriptions tend to concentrate on the lack of range rather than how such systems function in the grammar, and whether they should be compared with numerals at all, rather than how they fit into the study of comparative numeral syntax and semantics. An early exception is Hale [9], who argues that Australian restricted-range systems are not actually numerals, but rather quantifiers more akin to *few*, *some* and *many* than to 2, 3 and 4. We should also note that many descriptions of numerals in Australia generalize across languages, even on points where it is known that the languages differed (see [10] for examples and discussion). In writing this article, I repeatedly raise questions that cannot (yet) be answered, because of the focus on ‘restriction’ rather than on ‘grammar’, and the assumption of uniformity across restricted systems. Restricted-range systems lead to discussions of bases, anchors and numeral compositionality [11,12]). Can anchors be identified in restricted-range systems? Are they productively compositional, with limits arising because of unwieldy compounding? Or do they have ad hoc anchors?

I begin in §2 with an overview of restricted-range systems, whether it makes sense to discuss ‘restricted’ or ‘low-limit’ in contrast to ‘unrestricted’ systems, and whether such systems can be said to exhibit bases. In the remaining sections, I turn to three areas where we might compare numeral systems cross-linguistically: etymologies of numeral words in §3, the status of degrees in §4 and the lexical class of numerals and morphological number in §5. In the interests of clarity, I predominantly illustrate arguments with two languages, Bardi and Warlpiri.^2^

## 2. Restricted-range systems

### (a) Are all linguistic numeral systems ‘restricted’?

Greenberg’s [13] first generalization about numerals states that ‘every language has a numeral system of finite scope’. The basis of his argument is twofold: that natural languages lack terms for infinitely many numeral powers, and that Greenberg himself did not have a word to express numbers above 10^33^. This latter point has a rebuttal by Comrie [14], whose grammar recursively generates terms that Greenberg’s did not. Whether or not languages can express an infinite set of numerical items, it is clear that some languages have much lower limits than others.

Numeral systems might be restricted because the lexical set of numerical words is a closed set. That is, a system might be restricted because there is no lexical item to express numbers above a particular threshold. For English that might be 10^92816532^+1, for another language it might be 8. Lexical categories may be open or closed; closed classes do not permit new members, while open classes do. In English, the set of verbs is open: speakers can add new verbs grammatically. In Bardi [15] or Warlpiri [16], verbs are a closed set and new items cannot be added grammatically. Thus, one possibility is that in some languages numerals form a closed (and small) class, while in others the class of numerals is more open, analogously to colour term differences across languages, where some languages have a smaller, restricted set of terms while others use a larger and more productive range. This has not, to my knowledge, been tested.

Language users might be able to combine smaller numbers arithmetically to form larger ones, but with an upper limit constrained by the time needed to pronounce the forms. In that sense, all languages are indeed ‘restricted’. This seems to me to be an odd argument, however. After all, such considerations are not usually taken to be an indication of finiteness in natural language. The argument for infinite recursion, going back to at least Hockett [17], says that languages like English have potentially infinite recursive expression because the constraints on sentence embedding are physical (the time it would take to say the sentence), not grammatical. In (1), for example, there is nothing *grammatical* preventing a limitless chain of embedding. The same argument should apply to numbers.

**Table:** 

(1)	Alex said that Ben said that Carrie said that Derek said that Edgar said that …

If we are not looking at a lexical limit, we could consider a grammatical one. Compositional numerals are often compounds. Languages differ in what types of compounding are grammatical. Some languages allow free and recursive compounding (e.g. English or Finnish) while others have strict constraints on compounding. Bardi, for example, has only appositive compounds, and they occur in a limited number of semantic fields (for example, in kinship terms such as *birrii+gooloo* ‘parents’ (mother+father)). Moreover, compounding in Bardi is not recursive. Therefore, numerals might be restricted because of a lexical restriction on compound formation, rather than a restriction on the expression of numerical concepts. This has not, however, been tested across languages.

Whether the difference is primarily lexical or grammatical, I take there to be a difference that is worth exploring between numeral systems where theoretical maxima are in the billions or trillions, versus those where the largest expressed concepts are under 10.

### (b) Overview of Australian numeral systems

In this section, I provide an overview of the characteristics of Australian restricted-range systems. The following information on Australian restricted-range systems is summarized from my previous work [7,10,18]. Zhou & Bowern [18] use a sample of Australian Indigenous languages to infer evolutionary facets of restricted-range numeral systems. Bowern & Zentz [10] provide further details, based on a survey of 189 languages from both Pama-Nyungan and non-Pama-Nyungan families.^3^

The majority of Indigenous Australian numeral systems have restricted ranges. Not all such systems have the same limit, however. Zhou & Bowern [18] reconstruct 4 as the most likely limit for Pama-Nyungan numerals, but the limits in their sample range from 2 to 20. Tyapwurrung (Kulin, Pama-Nyungan) numerals are unrestricted, according to Dawson [20]. Three-quarters of the languages (139/189) had upper limits of 3 or 4. Several languages show more recently elaborated systems (e.g. Warlpiri, Gamilaraay), either with terms borrowed from English or terms created internally. Nine languages in the Bowern Zentz [10] sample had only words for 1 and 2, but in almost all cases, there is reason to believe that the upper limit may have been higher, and the absence of a recorded word for 3 (or additional numerals) relates to the scarcity of documentation [21].^4^ All languages have sequences of numerals: that is, 1, 2, 3, 4 rather than 1, 2, 4 (with no word for 3).

Some languages have numerals that are compounded from other numerals (that is, they are compositional); in other cases, each numeral is unrelated etymologically to others in the system. As seen in Bardi below, 1, 2 and 3 are opaque, while 4 is compositional (2+2):

**Table:** 

(2)	Bardi numerals [15]	
	*arinyji*	*1*
	*gooyarra*	*2*
	*irrjar*	*3*
	***gooyarragooyarra***	*4*

Zhou & Bowern [18] and Bowern & Zentz [10] discuss the distribution of opaque and compositional numerals in Pama-Nyungan. The two-thirds of the languages (117 of 177 languages with an attested numeral for 3) have an atomic (that is, otherwise unanalysable) 3. For the vast majority of compositional numeral 3s, the form is 2+1. There is a relationship between compositionality and numeral extent. Systems with only three numerals are more likely to have non-compositional numerals; if the system extends beyond 3, it is more likely that the numerals for 3 and above will be compositional. Some languages have both compositional and opaque numerals, such as Warumungu.

**Table:** 

(3)	Warumungu [21]		
	*kujjarra-yarnti*	*‘ *three’	2+1
	*yurrkarti*	*‘ *three’	

### (c) Anchors and composition in restricted-range systems

Anchors provide structure to a numeral system: if the system is compositional, there is a set of digits with position and the potential for combination to express arbitrarily large numerical concepts. A genuinely anchorless or non-compositional numeral system would be restricted by definition to the number of numerical lexical items. That raises the question about whether systems (*qua* systems) exist. We can investigate this in two ways: by considering how anchors are expressed in low-limit systems, and by comparing the syntax and semantics of restricted and unrestricted numeral systems in order to better understand how they differ. The first gives us evidence about compositionality, the second about systematicity.^5^

Several Australian languages could be argued to have systems built around either additive or multiplicative anchors. For 70% of the languages where a term for 4 is recorded, the term is either a transparent compound 2+2 or etymologically such a compound with subsequent sound change (e.g. Mudburra *kutyatyarra < kutyarra-kutyarra*); five languages have 2+2 terms alongside atomic terms. A few have terms which are compounds of 3+1.^6^ Terms for 5 tend to be based on words for ‘hand’; most of the other terms are either 4+1 or 2+3. Terms for 6 are rare and vary in composition: they include 5+1 (eight languages) as the most common pattern. Other patterns are found in only one or two languages: 2+2+2, 3+3, 2*3, 4+2. Two languages have non-compositional forms. One of these forms for 6 appears to have a multiplicative anchor (2*3 in Koko Bera), while others appear to be additive (3+3 or 2+2+2). Zhou and Bowern discuss reconstructions and regional patterns.

Whether or not there is evidence for bases, there is evidence that numerals have systematic behaviours and parts of the system build on each other. For example, there appears to be a strong dispreference for systems that have compositional lower numerals but non-compositional higher numbers (e.g. 3 expressed as 2+1 but atomic 4). That is, where there are compositional numerals, they are the larger members of the set.^7^ The numeral 2 is always atomic (never 1+1), which is unexpected if we assume that restricted-range systems are always compounds of smaller numerals. Most languages have no available data on variability. Thus in summary, there is some evidence for anchors in restricted-range systems, but the evidence is limited, and they do not provide a scale for building larger numerals. This raises the question of whether such systems are numerical but non-compositional, or whether they are not actually numeral systems at all as Zariquiey *et al.* [8] conclude for Pano languages. In order to investigate this question, in §3, I consider etymological sources of Australian numerals, while in §4, I discuss their syntax, semantics and comparison with other expressions of quantification.

## 3. Words for numerals

### (a) Etymologies of numerals

Numeral systems have three main etymological sources, as well as exhibiting atomic (underived) numerals without clear etymologies. The most common source of numerals in most languages is perhaps unsurprisingly, other numerals. Numerals are built on one another more or less systematically, with lexical anchors (e.g. [11]) that may be additive, subtractive or multiplicative [6,22]. Sound change may make the etymologies opaque (as in English *fifteen* < 5+10). A second source is borrowings from numerals from other languages (cf. [23]). A third is body parts, particularly ‘finger’ and ‘hand’. Finally, many numerals are reconstructable as underived lexical items back to a common ancestral language.

For restricted-range systems, Epps *et al*. [7] survey Australia, Northern California and Amazonia (see also [24] for Amazonia and [10] for Australian languages). In Australia, many numerals are underived and unetymologizable. Those that are derived come from, by and large, the same sources as non-restricted-range systems: other numerals (e.g. Wangkayutyuru *parrkulu-kunyu* for 3 (literally ‘two-one’), body parts (Bardi *nimarla* ‘hand’ for 5) or other languages (particularly English). Some languages have numerals that describe the shape of the Arabic written numeral, e.g. words related to ‘pannikin’ (cup) for 9 (evoking the shape of a cup with handle when viewed from above).

The word for 1 shows a link between words for *together* or *alone*, though the direction of semantic change is not clear.^8^

**Table:** 

(4)	Arabana [25, p. 104]
	***nguyu**-nga-ma*
	one-loc-make
	‘to put together’

### (b) Numerals and demonstrative etymologies

Etymologically, Australian systems appear to show similar sources to languages elsewhere in the world. There is one difference, however. There does seem to be an etymological connection between the numeral 2 and dual demonstrative pronouns. A form *pula is reconstructible as a numeral across a wide set of Eastern Australian subgroups, including Bandjalangic, Kulin, Waka-Kabi and Yuin-Kuri. As a 3dl form, *pula is found further west (in Karnic and Thura-Yura, as well as some of the subgroups that also have the form as a numeral). The most widespread 2 form is *kutyarra, which is also found as a dual number marker.

Numerals for 1 tend to be a source of indefinite articles in languages across the world: Romani [26], Amharic [27], Abkhaz and Danish, for example, as well as English *a(n*). This pattern appears to be rare in Australia, with only (at this stage) two languages reported with this usage: Bininj Gunwork [28] and Kayardild [29]. This is because articles in general are rare, not that indefinites have another etymological source.

### (c) Other words related to numerals

Numbers go along with other vocabulary, which is unfortunately largely unexplored in most languages. Words for counting are not frequently attested in dictionaries of Australian languages, but they do exist. The Bardi verb root -*joombar*- count appears to be reconstructible to Proto-Nyulnyulan (it is found in both branches of the Nyulnyulan family). A root *pinta* or *winta* is found in Karnic languages (a subgroup of Pama-Nyungan). A few languages use loans; Yolngu Matha *bothurru* ‘to count’ is from Macassar ‘play dice, gamble’^9^ and Nyamal and Yandruwandha both attest verbs derived from English *count*, e.g. Yandruwandha *kantama*.

There are ways of asking questions about quantity, e.g. translations of ‘how many’, in languages across the country (e.g. Pitjantjatjara *yaaltjiṯu*, Payungu *nhawara*, Warlpiri *nyajangu*, Wiradjuri *minyangga* and Yawuru *ngandja*). About 300 varieties are attested with a term translated as ‘how many’ or ‘how much’ in the Chirila lexical database [30].

**Table:** 

(5)	Baagandji [31, p. 170]	
	*N* *a* *nd* *ara*	*wimbargu-ama*
	how.many	children-2.sg.POS
	‘How many children do you have?’	

It is unclear if any Australian languages have a distinction between mass nouns and count nouns (which corresponds in English broadly to quantities ‘many’ versus ‘much’). Bardi does not, with *niimana* used in both contexts. Bowler & Kapitonov [32] argue that the distinction is probably absent, though they note that translations of ‘big’ are found as intensifiers in mass but not count noun contexts.

While the grammars and dictionaries that give translations for ‘how many’ do not typically give information on how such questions are answered, in the Yidiny grammar [33, p. 199] is explicit that the answer to such a question can be a quantifier (such as *ngabi* ‘a lot’) or a numeral. As Bittner & Hale [34] note, however, in Warlpiri *nyajangu* is ambiguous between a question about quantity (i.e. ‘how much’) and one that asks ‘what kinds’. That is, a possible answer is a listing of types as well as a statement of quantity. The same is true for Bardi *nyirroogoordoo*, etymologically ‘how+each’.

It is unclear whether Australian languages have paranumeric words equivalent to English words such as *dozen* or *gross*; that is, words that refer to quantities but which are not part of the counting system. Some languages have words referring to pairs (dyads), and there are birth order names up to 10 [35] in Thura-Yura. Finally, Australian languages have quantifiers (for which see [32]).

## 4. Numerical semantics

Numerals are their own linguistic subsystem ([36]; see also, among others [37–39]). Numeral concepts in linguistics are not the same as numbers in mathematics, as a number of other articles in this issue also make clear. For example, all languages have concepts of negation and absence (e.g. [40]) but the numerical concept 0 only enters mathematical reasoning in the twelfth century (e.g. [41] and earlier [42]).

Linguists have also made clear that numerals are not uniform in language. From different perspectives, Kayne [38] and Veselinova [43] present ways in which different parts of the numeral scale have different syntax and morphology. In this section, I focus on numerical semantics to investigate potential differences between low- and high-limit numeral systems.

A recurring issue for the linguistics of quantification, and many other aspects of grammar, is the notion of *degrees*.^10^ In semantics, degree functions are a formal mapping to express relationships between items that can vary by some amount. For example, in the phrase ‘chickens are much larger than squirrels’, we have an entity (*chickens*), a comparison class (*squirrels*), a property (*largeness*) and a degree modifier, the extent to which chickens are larger than squirrels (*much*). All languages appear to have ways of expressing comparisons, but they differ extensively: whether the comparison is explicit or covert (inferred through implication), in the amount of precision that is expressed in such constructions, along with the syntactic constructions used. Degree functions are used to analyse measurement phrases, comparatives and sometimes enumerative uses of numerals, as discussed further in the following sections. Degree constructions are thus a critical area to examine numeral systems cross-linguistics.

### (a) Degrees and enumeration

Numerals are commonly used for enumeration of cardinalities—that is for counting the number of objects, as in English sentences such as (6)

**Table:** 

(6)	There are **three cats** on the windowsill.

Numerals can be used in measurement and in providing precise comparisons, as in (7) and (8), respectively.

**Table:** 

(7)	Add **three cups of flour** to the other dry ingredients.

**Table:** 

(8)	Anna is **three inches taller** than Zak.

Numerically, all these examples count some amount (cats, cups of flour or the degree to which someone is taller than someone else). Semantically, however, enumeration and measurement have been given distinct semantic analysis. Snyder [53] traces this view back to at least Frege [54], where counting involves identifying the cardinality of a set (that is, providing an integer answer to the question of ‘how many’ of an item^11^), whereas measurement phrases ‘assign a quantity on a dimensional scale’ [55, p. 5]. That is, measurement (as in (7)) entails a scale with degrees, whereas counting involves enumeration of the elements in a set: measurement of ‘dense scales’ versus discrete collections of individuals, as Fox & Hackl [44] put it. An alternative view is proposed by Fox Hackl [44] and Snyder [53], where there is no distinction between discrete enumeration and measurement, and all scales are dense. This analysis solves a number of contradictions in the two-types approach. It does imply, however, that all numerical measurement (discrete and otherwise) involves degrees on a dimensional scale. That is, it makes use of the degree semantics described above.

The two-types approach may have an advantage if languages distinguish enumeration from measurement (e.g. allowing sentences of the type exemplified in (6), while disallowing (7) and/or (8). Languages differ in the extent to which they express these different types of measurement phrases and appear to make use of degrees in their semantic ontologies. Languages such as Washo [56], Motu [52] and the Australian (Pama-Nyungan) language Warlpiri [57] are argued to not introduce degree functions to the grammar.^12^ Degrees as a linguistic primitive are central to many analyses of comparative constructions, gradable^13^ adjectives and negative polarity, among other aspects of syntax and semantics. That is, degree functions are integrated into formal analysis of areas of grammar far beyond enumeration, and so requiring cardinal numerals to instantiate a measure function is a strong claim that reaches far beyond the grammar of numbers. If a language does not make use of degrees in the semantic ontology, yet has numerals which otherwise behave syntactically and semantically like numerals in degreeful languages, an obligatorily degreeful analysis is problematic. Conversely, degreelessness may be related to the restricted-range numeral systems, and perhaps compositionality is what provides linguistic evidence for degrees.

### (b) Degrees and measurement in Australia

Numerals in Australian systems are attested primarily in contexts of enumeration. These are the most common (and sometimes the sole) examples in reference grammars and dictionaries.^14^ An example from Bardi is given in (9).

**Table:** 

(9)	Bardi	
	*gooyarra*	*minyaw*
	2	cat
	‘Two cats’	

Measurement phrases seem to be mostly ungrammatical, implying an absence of degree constructions. Bowler’s [61] discussion for Warlpiri is one of the few detailed investigations where such items are systematically either code-switched to English or rephrased. The situation is less clear for Kunbarlang (see [60]). The closest is this elicited Bardi phrase in example (10) below. However, this is better analysed as appositive: ‘give me two cups, [give me] sugar’.

**Table:** 

(10)	*Gooyarra*	*banigin*	*a-n-a=ngay*		*jooga*	
	2	cup	2-tr[give]=fut=1sg		sugar	
	Intended: ‘Give me two cups of sugar.’					
	Literal: ‘Give me two cups … sugar.’					

A few Bardi examples also involve time measurement, but such examples could probably also be analysed as enumerations rather than measurements. Measure phrases are absent from comparative constructions, which themselves are rare in Australian languages [62].

**Table:** 

(11)	Bardi [15, p. 577]				
	*Biindan*	*gooyarra*	*booroo*	*i-nga-moolgoo-n*	
	scrub	2	time	3-pst-sleep-cont.	
	‘He slept in the scrub for two days.’				

Given that measure phrases (and other degreeful constructions) are generally absent in Australia, it is tempting to conclude that this is related in some way to the structure of the numerical systems. However, we cannot simply say that degree semantics and low-limit numerals are closely related, as some Oceanic languages, including Motu and Samoan, are said to lack (or have recently acquired) degree semantics but have larger sets of reconstructable numerals.

## 5. Numerals and lexical class

The previous section introduced the concept of degrees and raised the question of whether the non-universality of degree constructions could relate to whether numerals are compositional, and whether they are linguistically best analysed with degrees or whether there might be some languages where numerals introduce cardinality without an ordered scale. Another point of theoretical interest is how numerals relate to other quantificational marking in language.

### (a) Are numerals quantifiers or adjectives?

Number is one system of quantification in natural language. There are also quantifiers (English *some*, *many*, *all*; Bardi *niimana* ‘many/much’, *boonyja* ‘all’) and morphological number marking in nouns, verbs and pronouns (typically singular/plural, with dual, trial and paucal systems also attested; see [63]. English has numerals, but it also has quantitative concepts such as *pair*, *couple*, *few*, *some* and *many*. Another area of theoretical interest is whether numerals are adjectives or quantifiers (that is, a type of determiner). That is, do numerals restrict reference (like adjectives such as *good* or *tall*)? Or do they quantify entities, more like words such as *many,* or like morphological plural marking [64]? Perhaps restricted-range systems are best analysed as a system of quantificational determiners.

Bylinina and Nouwen [65] provide a summary of the arguments for each position, focusing on data from English. On the one hand, numerals pattern like quantificational determiners such as *some* and *every* from the perspective of substitution tests. Given that the function of numerals is to quantify, this is at first glance reasonable.

**Table:** 

(12)	These/Some/Twelve cats sat on the windowsill.

In other ways, however, English numerals are not like determiners, but more like adjectives. Determiners cannot be doubled, but a numeral and another determiner can co-occur, just as determiners and adjectives co-occur:

**Table:** 

(13)	a. The twelve/fluffy cats sat on the windowsill.
	b. *The these cats sat on the windowsill.

Another puzzle, as Bylinina & Nouwen [65] note, comes from sentences like the following (their example 6):

**Table:** 

(14)	Twelve apples can fit in this shoebox.

Such sentences involve, as they say, quantifying over groups rather than enumerating specific items. Their paraphrase is ‘any normal group of twelve apples is such that this group fits in the shoebox’. In that way, numerals are like adjectives: compare ‘green apples can fit in this box’—any normal group of green apples is such that it will fit.

A final contrast between numerals and quantifiers involves collective readings:

**Table:** 

(15)	a. Five cats fit in the cardboard box.
	b. Every cat fits in the cardboard box.

In (15b), the most salient reading is that for each cat (individually), the box is big enough to hold it, not that all cats in the world can be placed at once in a box. That is, a quantifier like *every* describes properties common to each individual, while a numeral word like *five* provides information about the size of the group denoted by the plural noun. In that sense, numbers provide adjectival information about cardinal quantities, in the same way that colours provide adjectival information about hue. Therefore, while English numerals have some properties shared with quantifiers, in other ways, they are more similar to adjectives.

In languages like English, quantifiers such as *some* and *many* are clearly distinct from numerals. However, restricted-range numeral systems, in some ways at least, look more similar to quantifiers. Hale’s [9] view of Warlpiri numerals was that they are more like quantificational indefinite determiners with number marking than English numerals, and that they correspond most closely to a set of number-marked definite versus indefinite determiners. That is, for Hale, the proper analogue of Warlpiri words like *jirrama* is not English ‘three’, but rather English ‘some’.^15^

**Table:** 

(16)	Warlpiri		
	Indefinite		Definite		
	*jinta*	‘singular, one’	* nyampu*	‘this, singular’	
	*jirrama*	‘dual, two’	*nyampujarra*	‘these, dual’
	*wirrkardu*	‘paucal, several’	* nyampupatu*	‘these, paucal’
	*panu*	‘plural, many’	* nyampurra*	‘these, plural’

Hale is led to this conclusion in part because Warlpiri numerals can be used with vague reference (that is, *wirrkardu* is often given as a translation of English ‘three’, but unlike English 3, *wirrkardu* can be used with approximate quantities. The quantifier analysis neatly accounts for restricted-range systems where numerals have vague readings, but makes additional predictions which are problematic (discussed further below).

### (b) Numerals as adjectives

An alternative to the quantifier approach is to analyse numerals as adjectives. While Australian languages have sometimes been argued to lack clear distinctions between nouns and adjectives, in a review of this question Kim [66] surveys such constructions and finds about half the languages of the country appear to show consistent evidence for a distributional distinction between words denoting entities and those denoting properties. That is, in most—but not all—cases where the question has been explicitly tested, there is evidence for a lexical class of adjectives that is distinct from nouns. That is, there is a difference between Warlpiri, where property concepts appear to be nouns and there is no separate class of adjectives, and m Bardi, where there is a distinction (see [15]).

In Bardi, at least, there is some evidence that numerals are adjectival. They can co-occur with demonstrative pronouns, for example. Numerals and other adjectives can appear in either order modifying a noun.

**Table:** 

(17)	Bardi [15, p. 295]			
	*Irr*	*irrjar*	*barnmiidan*	*ingarr-jarrala-na*
	3pl	3	that.way	3pl-run-rem.pst
	‘Those three [people] ran that way.’			

**Table:** 

(18)	Bardi		
	a. *gooyarra*	*goolarr*	*maalba ~*
	2	small	baby
	b. *goolarr*	*gooyarra*	*maalba*
	small	2	baby
	‘two small babies’		

Given this difference, it’s quite possible that some languages have numerals which pattern with property concepts (like Bardi) and some where they are more properly analysed as quantifiers. Louagie [67] provides further discussion and suggests that different numeral analyses may be needed. Word class is therefore inconclusive for numeral analysis (or rather, any analysis that requires numerals to be quantificational determiners or adjectives will not cover the phenomena we have evidence for). Likewise, while it may be tempting to treat low-limit systems as determiners, that analysis will not hold across the board either.

### (c) Numerals and approximate reference

One aspect of restricted-range numbers that has received some attention is that in at least some languages, their reference can be to approximate rather than to precise quantities. However, it is fairly easy to find examples of numerals in unrestricted systems where a numeral is used but the interpretation is felicitous with an imprecise valuation of that number. This reflects Lasersohn’s concept of the ‘pragmatic halo’ [68,69], where pragmatic concept allows felicitous interpretations from utterances that should technically be impossible. Consider (19), for example:

**Table:** 

(19)	I must have passed **ten** of my students on the way to work this morning.

Such a sentence is licit even if I passed only nine students. Clearly, this stands in distinction to the numerical expression 5+5, which cannot sometimes equal 9 and sometimes 10. That is, numerals pick out concepts, not exact numbers of referents in sets. That is, I do not consider approximate reference to necessarily be a diagnostic for differences in numeral behaviour across languages.

Numbers can be pluralized, which alters their reference. For example, if ‘hundred’ is pluralized to ‘hundreds’, it does not necessarily mean a multiple of one hundred. Information about usages like this in restricted-range systems is lacking.

**Table:** 

(20)	I dropped a jar and spilled **hundreds** of beads on the floor. There were **879**.

Larger numerals in English can have vague reference; smaller quantities, however, do not. The extent to which numerals have vague reference in Australian languages appears to vary. In Warlpiri, for example, *wirrkardu* can translate English ‘three’, but does not exclusively translate it. In Bardi, in contrast, numerals themselves do not have vague reference, they denote exact quantities. To signal an inexact number, quantifiers such as *jalboor* ‘few’ can be used, or numerals in apposition:

**Table:** 

(21)	Bardi [15, p. 282]			
	*Ginyinggon=min*	***gooyarr***	***irrjar***	*arra-loong-an=irr.*
	then=LINK	2	3 1pl-collect-CONT-3PL.DO	
	‘Then we collect two or three more.’			
	*Then we collect five more			

While vague-reference numerals like Warlpiri’s may well suggest an approximate quantifier analysis, the variation in reference across restricted-range systems means that we cannot rely on such an analysis for understanding these systems in general.

### (d) Numerals as indefinites and morphological number

Some Australian languages numerals appear to have approximate reference for at least some numerals. Given that, the claim that numerals might in fact be co-extensive with morphological number features is at first sight plausible. Another piece of evidence comes from etymology; one of the reconstructed forms for the numeral 2, *pula, is very similar in form to dual demonstrative forms, and there seems to be a close relationship between dual marking and words for 2, as discussed in §3.2.

This is a testable hypothesis. For example, indefinite determiners cannot pick out specific individuals. If Warlpiri *jinta* is an indefinite singular determiner rather than a numeral, it should not be able to appear in contexts such as the following:

**Table:** 

(22)	I saw a dog and a cat. #A cat was meowing.

Bittner & Hale [34, p. 96] give the following readings for an example sentence with Warlpiri *jinta*:

**Table:** 

(23)	*Jinta*	*ka-rna-ø*	*nya-nyi*
	one	PPRES-1s-3s	see-N.PST
		I see one (of them).	
		I see the one.	
		I see him/her/it, which is one (i.e. alone).	

All these examples are definite, and in fact, Bittner & Hale argue that Warlpiri phrases with ‘numerals’ have both definite and indefinite readings (though they do not provide examples in the discourse context of (17), which is crucial). This absence of information is unfortunately too common for documentary semantics for Australian languages.

Even if the indefinite determiner analysis of *jinta* is not correct, it may still be tempting, particularly for restricted-range systems with low non-compositional units, to treat the ‘numerals’ as morphological number features: singulative, dual and plural (or paucal and plural). This, as in the determiner claim, is an empirical claim that requires substantiation. Do they occur in the same environments? Such an analysis may work for highly restricted systems (1, 2, 3+), but it is unlikely to be plausible for large limit-restricted ranges, simply because quantifiers across the world’s languages tend to instantiate only a few levels of quantification (*few*, *some*, *many*, for example, with three levels). It also does not explain how ‘quantifiers’ can appear as anchors (like Bardi *gooyarra* ‘two’), even ad hoc ones.

If Australian language numeral systems are actually plurality systems (as claimed, for example, by Hale), they should behave more like morphological number systems grammatically. A further prediction for this analysis may be that languages should have ‘singular’ and ‘plural’ numbers, but not ‘dual’ or ‘paucal’ numbers (following the morphological universal that singular~dual~plural systems emerge from singular~plural ones). But we do not see such a system: in the Bowern & Zentz [10] sample, all languages had sequences of number words (1, 2, 3), not a ‘one-like’ word and a ‘four-like’ word. This stands in contrast to morphological number category universals. Language have singular–plural or singular–dual–plural distinctions; not singular–dual versus singular–dual–plural.

Concepts may be grammatically plural but conceptually singular. The noun ‘trousers’, for example, is conceptually singular but morphologically appears with plural marking. Likewise, other *pluralia tantum* such as *scissors*. This is in contrast to a notion of plurality occurring with items that are more than one.

**Table:** 

(24)	Pass me the **scissors**.

*Pluralia tantum* are found in at least some Australian languages. In Bardi, for example, *gaalwa* ‘mangrove raft’ takes plural verb agreement [15] even when only one raft is under discussion. If Australian numeral systems are more like morphological number, we might expect that numerals may be used in such contexts. This has not, to my knowledge, been reported (but may not have been tested).

### (e) Numerals and paranumerals

Finally, productive numeral systems can coexist alongside reference to specific quantities (e.g. a *gross* alongside ‘one hundred and forty-four’; a *pair* alongside ‘two’). These items are sometimes substitutable for numerals. In other cases, however, they have different syntax. The following pairs of sentences illustrate some of the differences.

**Table:** 

(25)	a. Please buy a gross of nails.	
	b. *The giraffe is taller than the lion by a gross of/three dozen centimetres.	

**Table:** 

(26)	a. There are two dozen more bagels on that shelf than this shelf.	
	b. *He beat me to the finish line by a gross of seconds.	

**Table:** 

(27)	a. How many donuts do you have?	
	b. one/two dozen	
	c. *zero dozen (cf. zero)	

**Table:** 

(28)	a. I’ll have half a dozen eggs.	
	b. *I’ll have half [a] twelve eggs.	

**Table:** 

(29)	a. Two pairs of shoes		(= 4 shoes)
	b. ?A pair of two shoes		(= 2 shoes)

A possible analysis for restricted-range systems might be that they are sets of paranumerals: that is, items like ‘dozen’ rather than numerals like 12. No one to my knowledge has investigated this question in detail for Australia, but I suspect that Australian numerals are not paranumerals. First, these items do not occur in sequences, but refer to particular quantities (such as 144, a ‘grosse douzaine’ or ‘big dozen’ (12^2^)). Second, they tend to occur with some types of items but not others (that is, they have a classificatory component), as shown in (28-29). This is not found, to my knowledge, at least in Bardi.

## 6. Conclusions

To return to the questions posed in §1, Australian restricted-range numeral systems come from the same sources as numerals in other languages (that is, loan, other numerals and body parts). Grammatically, they behave in many ways like numerals in unrestricted systems: they enumerate concepts and show the same variation in co-occurrence with morphological plurality. For at least some languages, they behave morphologically like adjectives (rather than like quantifiers or determiners). In other ways, Australian restricted-range numeral systems are quite different. They do not, for example, participate in measure phrases.

Restricted-range systems are not uniform, however. They vary in limits and in vague reference, for example. This causes problems for analyses that equate restricted systems with quantifiers. It also raises questions about analyses that treat such numerals as morphological plurality. Not least, plurality systems with singular/plural distinctions are much more common than those with duals or paucals, but restricted-range systems with limits around 3 (or 4) are the norm.

Many Australian numeral systems show at least limited compositionality, with anchors of 2 or 3. This compositionality makes it less likely that Australian numerals are quantifiers or equivalent to determiners with morphological number marking.

Because these languages do not grammaticalize degrees, they cast doubt on analyses of numerals that require degreeful grammars. However, degreefulness and unrestricted ranges are not deterministic, since there are unrestricted numeral systems in languages with degreeless grammars. Restricted-range systems give an interesting opportunity to examine the different ways in which instantiations of number marking—plurality, numerals and quantifiers—are marked grammatically and contribute to linguistic meaning.

In §2, we asked why restricted-range systems might be restricted: whether it was because they are non-compositional, lack anchors or are a different type of quantification from unrestricted systems. On the available evidence, it is not possible (yet) to answer this question fully. Restricted-range systems as attested in Australia do not lack anchors, but they do appear to not have positionality. It is unclear, however, whether this is a morpho-lexical restraint on compound size, or a feature of the numeral system itself.

## Data Availability

All data mentioned in this article have been published elsewhere and are cited in the text.

## Footnotes

^1^
See also Epps *et al*. [7] for an overview of numeral systems in hunter-gatherer languages, not all of which are restricted range. However, Australia had the largest proportion of restricted-range systems in Epps *et al*.’s sample. Zariquiey *et al*. [8] discusses restricted-range systems in the Amazonian Pano languages, where it is clear that there are several differences from the Australian languages discussed here (e.g. in the conventionalization of numerals above 2, and the explicit marking of quantification).

^2^
Throughout the article, I use Arabic numerals (1, 2, 3) to represent numerical concepts, and spelled-out words (*one, two, three*) to represent lexical items.

^3^
Another source of information about Australian numerals is Hanke [19], but there are enough errors in the Australian data (as discussed by Bowern & Zentz [10]) that we use Australian-specific sources for discussion here.

^4^
An additional complication is whether words for 5 based on ‘hand’ are numerals or an ad hoc method of describing an amount (see [8] for discussion).

^5^
We can make such comparisons within languages as well. For example, English *two* and *pair* refer to the same quantity of items (‘two oranges’ is the same amount as ‘a pair of oranges’) but syntactically and semantically they differ in numerous ways. For example, ‘two pairs of oranges’ is grammatical, but ‘*a pair of two oranges’ is not (* denotes an ungrammatical utterance). Using such tests we can examine whether numerals (e.g. translations of ‘two’) have the same properties across languages.

^6^
In this article,+ denotes additive compounding, − is a hyphen between morphemes, and < is used in its historical sense to denote an etymological source.

^7^
Compare Russian with compositional 39 but atomic 40 сорок *sorok*.

^8^
A broader range of etymological sources is found in South American languages; see Epps *et al*. [7].

^9^
The source is the online Yolngu Matha dictionary: https://yolngudictionary.cdu.edu.au/word_details.php?id=1288

^10^
For further information see, among many others, [44–51]. For languages that are argued to lack degree functions, see [52] and subsequent literature. There appears to be some ambiguity in the literature about degrees and scales. Here, scales are a general expression of some relation between two items on some dimension, where degrees are a type of scale which allows language users to identify precise points which are ordered in magnitude with respect to one another. As one might imagine, degree constructions are closely conceptually connected to numerals because numerals form a scale of just this type.

^11^
A perhaps counterintuitive consequence of this view is that (as far as I can see) there is no inherent linguistic ordering relationship between numbers. That is, the fact that 2, 3 and 4 form a scale is a mathematical fact, but not a linguistic one. In the degree approach, the scalar relationship is built-in.

^12^
The precise formalization of degree functions is beyond the scope of this article, but work in semantics treats degrees as a functional primitive in semantics. See further Kennedy [51] and for universality, von Fintel & Matthewson [58] ([52,56,59] are also relevant). See also Kapitonov [60] for discussion of degree semantics in Kunbarlang.

^13^
Gradable adjectives are those, like *tall* or *happy*, which can be differentiated on a scale and can take comparative and superlative forms, as opposed to absolute adjectives such as *prime [number]*, which cannot.

^14^
We cannot conclude from the paucity of examples of other types of construction that enumeration is the only use of numerals, however.

^15^
Note that Bittner & Hale [34] later argue that Warlpiri lacks determiners, and that words like *panu* can be either definite or indefinite. That is, they do not retain the analysis proposed for (16). I include this material here, however, since the view of numerals as indefinite determiners has gained traction in Australia. See also Zariquiery *et al.* [8] for some similar arguments for Amazonia.
